# Twin Boundaries merely as Intrinsically Kinematic Barriers for Screw Dislocation Motion in FCC Metals

**DOI:** 10.1038/srep22893

**Published:** 2016-03-10

**Authors:** Jiayong Zhang, Hongwu Zhang, Hongfei Ye, Yonggang Zheng

**Affiliations:** 1 State Key Laboratory of Structural Analysis for Industrial Equipment, Department of Engineering Mechanics, Faculty of Vehicle Engineering and Mechanics, Dalian University of Technology, Dalian 116024, P. R. China

## Abstract

Metals with nanoscale twins have shown ultrahigh strength and excellent ductility, attributed to the role of twin boundaries (TBs) as strong barriers for the motion of lattice dislocations. Though observed in both experiments and simulations, the barrier effect of TBs is rarely studied quantitatively. Here, with atomistic simulations and continuum based anisotropic bicrystal models, we find that the long-range interaction force between coherent TBs and screw dislocations is negligible. Further simulations of the pileup behavior of screw dislocations in front of TBs suggest that screw dislocations can be blocked kinematically by TBs due to the change of slip plane, leading to the pileup of subsequent dislocations with the elastic repulsion actually from the pinned dislocation in front of the TB. Our results well explain the experimental observations that the variation of yield strength with twin thickness for ultrafine-grained copper follows the Hall-Petch relationship.

Nanotwinned metals have attracted extensive research interest in recent years due to their unusual combination of ultrahigh strength and excellent ductility with little strength-ductility trade-off^1^,^2^,^3^,^4^ which makes them surpass their coarse-grained counterparts. The strengthening and toughening effects of the nanoscale stable coherent twin boundaries (TBs) have been extensively studied by experiments^1^,^2^,^3^,^4^,^5^,^6^,^7^,^8^,^9^, atomistic simulations^10^,^11^,^12^,^13^,^14^,^15^,^16^,^17^,^18^ and systematic investigations with the crystallographic analysis^19^. TBs can act as strong barriers to dislocation movement, resulting in hardening effects at the early stage of plastic deformation, or serve as emission sources of dislocations when the TBs gradually lose coherence^9^,^11^,^18^,^20^ due to residing sessile partial dislocations during further plastic deformation.

The strengthening effect is attributed to the dislocation interaction with and accumulation at TBs^17^, including the long-range static repulsion before the leading partial enters the TB and the kinematic impediment caused by the discontinuity of slip planes across the TBs. Though a lot of work has been done about the activation or strengthening mechanism, there are few quantitative studies about the static repulsion and kinematic impediment, especially their role in strengthening the material at different deformation stages, which is the cornerstone for predicting and designing the mechanical properties of materials with TBs.

In this paper, we first studied the intrinsic interaction between screw dislocations and TBs based on atomistic simulations and continuum models. In contrary to many previous researches^21^,^22^,^23^,^24^, both discrete atomistic simulations and continuum based analyses in this work suggest that the long-range interaction between a screw dislocation and a TB is actually negligible. However, the strengthening effects caused by TBs observed in experiments were usually attributed to the ‘strong’ repulsion between TBs and dislocations. Here by studying pileup behaviors of screw dislocations due to the presence of TBs, we reveal the actual origin of the observed strengthening effects and the corresponding results agree qualitatively with former experimental observations^1^,^25^. The new insights into the nature of TBs induced strengthening effects will considerably simplify the development of mesoscopic/macroscopic theoretical models that take into consideration the effects of TBs on the mechanical properties of materials.

## Results

### Energy variation of infinitely large TB-screw dislocation systems

**Figure 1:**
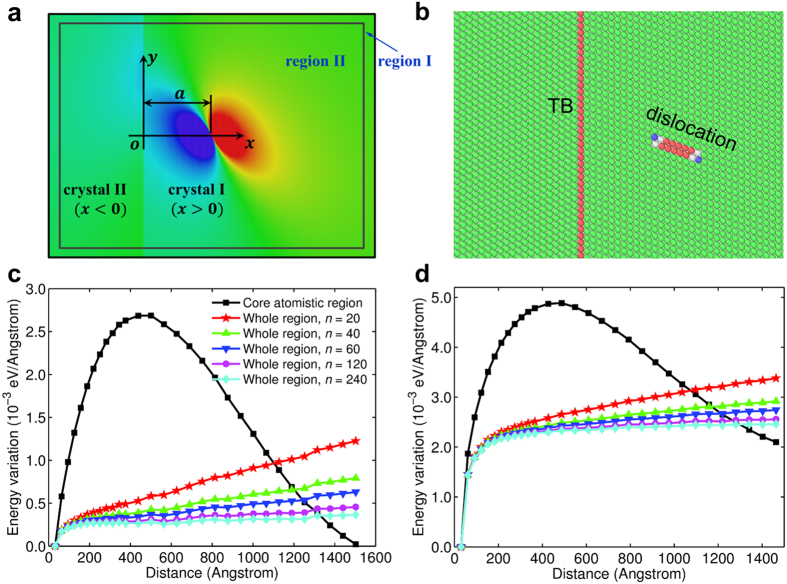
Coupled atomistic and continuum-based analysis: model and results. **(a)** Schematic representation of the coupled model. **(b)** The local configuration when the TB-dislocation distance is about 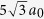. **(c,d)** The energy variation of the system as a screw dislocation moving away from the TB: **(c)** copper and **(d)** nickel. The lines with black square marks represent the results of the inner region II calculated with the atomistic method, while others show the sum of the energy of the inner atomic region II and the surrounding continuum region I with different thicknesses.

To investigate the intrinsic interaction between a single screw dislocation and a TB, we first examine the energy variation of infinitely large systems containing a screw dislocation and a TB. Here, coupled atomistic and continuum-based analysis is conducted based on the model shown in Fig. 1a. Similar to the idea of the CADD algorithm^26^ in the multiscale plasticity modelling, the model is composed of two parts: the inner region II represented by discrete atoms and the outside region (including the transition pad marked as region I) represented by continuum media. The displacement field of a screw dislocation in an anisotropic bicrystal^27^ based on the linear elasticity theory is applied to the discrete atoms and the continuum. The atoms in region II are then fully relaxed with the atoms in region I being fixed with prescribed displacements calculated based on the continuum theory.

The energy variation of the system, which is defined as the total energy of system (i.e., the sum of the potential energy of the inner core region II calculated with the atomistic method and the strain energy of the outside region calculated with the continuum method) with the dislocation located at (*a*, 0) subtracting the energy of the corresponding system with the dislocation located at 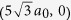, as a function of the distance between the TB and dislocation, is shown in Fig. 1c,d for copper and nickel, respectively. Conceptually, only in the case of infinitely large region – with no effects from the other boundaries besides the TB, the variation of strain energies with different dislocation-TB distances can be seen as the results of the intrinsic interaction between the dislocation and the TB. However, according to the classic elasticity theory of dislocations^28^, the strain energy of a straight dislocation in an infinitely large region is infinite (a logarithm function of the characteristic size), and that makes the numerical analysis unfeasible. Here, the size of the coupling atomic and continuum region is large but not infinite, i.e., (*n* · *L*_*x*_) × (*n* · *L*_*y*_) × *L*_*z*_, where *L*_*x*_, *L*_*y*_, *L*_*z*_, are the sizes of region II, and the size effects of the outer region are studied with *n* = 20, 40, 60, 120 and 240. We can see that as *n* increases, the slope of curve becomes smaller and the overall variation of the system energy (per unit thickness) is on the order of 10^−3^ eV/Å over a distance of about 1500 Å.

### Boundary effects on the energy variation of finite large systems

**Figure 2:**
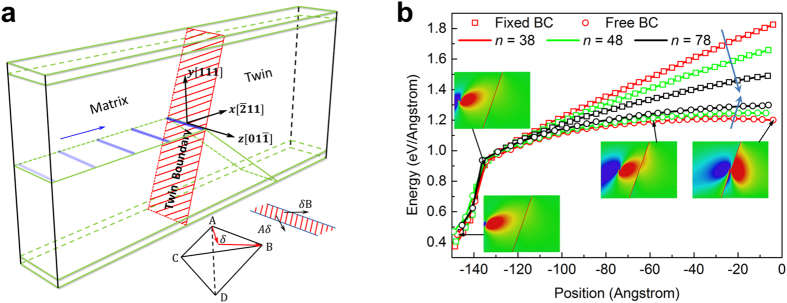
Minimum energy path for a screw dislocation approaching a TB. **(a)** Schematic representation of the simulation cell with the Thompson tetrahedron showing the crystallographic orientation of the matrix. A complete screw dislocation is dissociated into two partials. The blue arrow indicates the approaching of a screw dislocation to a TB and the long and narrow blue belts schematically represent the location of the screw dislocation in several intermediate states along the minimum energy path. The regions enclosed by green lines are the top and bottom boundaries. **(b)** MEPs for systems with different boundary conditions and heights in the *y*-direction. The zero-energy point represents the corresponding fully-relaxed dislocation-free cell. The insets show several representative configurations along the MEPs, which is color coded by the shear stress *σ*_*yz*_ according to the Virial stress formula which is equivalent to the Cauchy stress in an average sense.

In nanostructured materials, such as ultrafine grain materials, nanowires or nanoparticles, the free boundaries or common grain boundaries (GBs) may also influence the behavior of dislocations near TBs. In general, to a single crystal grain, the effect of other grains is equivalent to some kind of spring boundaries. Here, we investigate two extreme cases, i.e., rigid boundaries and free boundaries. The model is shown in Fig. 2a. A TB cuts the cell into two parts of equal size. The upper and lower boundaries are set free or held fixed with initial equilibrium displacements. Cells with different heights are investigated to study the effects of boundaries. Nudged Elastic Band (NEB) method^29^, which is one of the atomistic reaction pathway calculation methods based on transition state theories, is used to find the minimum energy path (MEP) for a screw dislocation moving towards a TB. Further details about the simulation can be found in the Method part.

Figure 2b shows the MEPs (i.e., the related potential energy per unit thickness as a function of the position of the screw dislocation) of the screw dislocation approaching the TB for systems with different boundary conditions and different heights in the *y*-direction. It can be seen from this figure that there is a knee point in each energy curve, representing the transition between two distinct processes: the steep part of the energy curve corresponds to the process of importing an extended screw dislocation from the left free boundary and the following part represents the motion of the dislocation moving from the free boundary to the TB. The activation energy of the first process is much larger, but it is not strongly dependent on the height of the model.

The energy increases in the samples with fixed boundaries are larger than that in samples with free boundaries. However, as the sample height becomes larger, there is a tendency for the energy curves of systems with different top and bottom boundary conditions to coincide with each other. It can be inferred that the coincidence of the two curves will occur in the case of infinite cell height as the influence of boundaries in the *y*-direction becomes negligible.

### Pileup of screw dislocations in front of a TB

The pileup of dislocations at obstacles, such as GBs, immobile dislocations or precipitates of a second phase, occurs at the early stage of plastic deformation. Materials with high strength can either hinder this pileup behavior to occur by reducing the grain size, or resist the stress concentration at the obstacles caused by the pileup of dislocations, for example, by introducing particles of a second phase. The pileup of screw dislocations in front of a TB is studied with a cell similar to, but larger than, that in Fig. 2a. External stress field *σ*_*yz*_ is imported by applying additional forces in the *z*-direction to the atoms in the upper and lower boundary layers. After introducing the dislocations one by one, the cell is fully relaxed by alternative energy minimization and dynamic relaxation.

**Figure 3:**
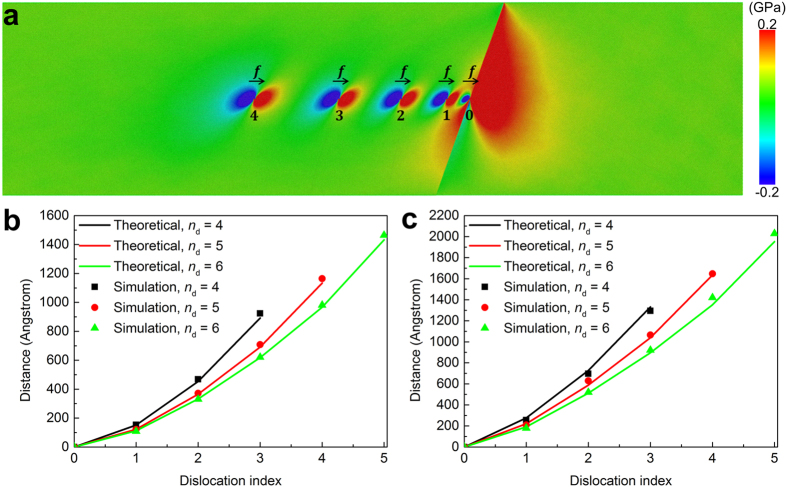
The equilibrium position of pileup dislocations. **(a)** Screw dislocations pile up against a TB. The symbol *f* represents the Peach-Koehler force due to the applied shear stress. Atoms are color coded by the atomic shear stress *σ*_*yz*_. The leading partial of the head dislocation has entered the TB, with corresponding stresses released in the twin crystal. **(b**,**c)** Comparisons of equilibrium distances from both elasticity theory predictions and atomistic simulations for copper and nickel, respectively. The vertical axis represents the distance from the *i*-th dislocation to the head dislocation (marked as 0). The black, red and green lines correspond to the pileup of 4, 5 and 6 screw dislocations, respectively, with stress *σ*_*yz*_ = 48.64MPa, 48.57 MPa and 43.86 MPa for copper and 41.55 MPa, 43.20 MPa and 42.41 MPa for nickel.

The equilibrium pileup configuration of five screw dislocations is shown in Fig. 3a with the atoms colored by the atomic stress *σ*_*yz*_. Stress concentration due to the severe distortion can be seen around a dislocation core. The leading partial of the head dislocation enters the TB with corresponding stress released in the twin crystal. However, the leading partial itself is not mobile in the twin crystal and cannot move along with TB either due to the constraint of the stacking fault ribbon connecting it with the trailing partial in the matrix crystal. Previous studies have shown that the two partials need to constrict into a complete one before redissociation into two partials in the twin crystal or in the TB^12^. The high stress caused by the pileup will promote this constriction-redissociation process.

The equilibrium positions (the central position of the stacking fault ribbon of each dissociated dislocation) for the pileup of 4, 5 and 6 extended screw dislocations for copper and nickel are shown in Fig. 3b,c, respectively. Moreover, the prediction of the equilibrium position based on elastic theory is also given by the solid lines. Actually, the pileup behavior of dislocations in front of TBs has been observed with transmission electron microscopy^30^,^31^,^32^. Especially, Chassagne *et al*.^32^ has observed a pileup of eight dislocations before transmission across the TBs under an applied stress 30–50 MPa in copper and the measured average dislocation spacing is about 30–40 nm. These experimental observations are qualitatively consistent with our simulation results, in which the average dislocation spacing is about 30 nm for a pileup of 4–6 screw dislocations under an applied stress of 40–50 MPa (see Fig. 3b,c).

## Discussion

**Figure 4:**
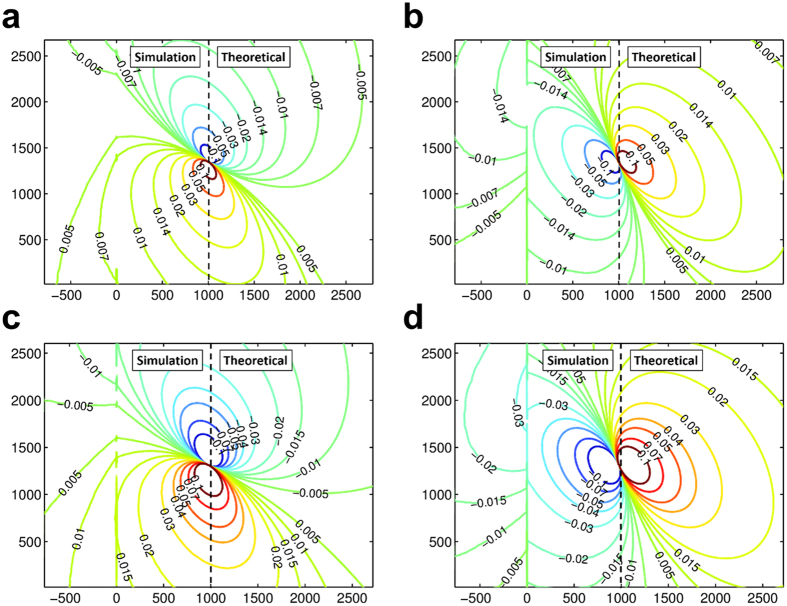
Comparison of the distributions of stress components *σ*_*xz*_ and *σ*_*yz*_ around a screw dislocation in a twinned bicrystal. **(a)** *σ*_*xz*_ for copper, **(b)** *σ*_*yz*_ for copper, **(c)** *σ*_*xz*_ for nickel, **(d)** *σ*_*yz*_ for nickel. The contour figures are divided into two parts by a dashed line: The left side represents the atomistic simulation results and the right side represents the results of anisotropic elasticity theory. The stress values in the contour plots are in the unit of GPa and the position is in Angstrom.

The critical issue in multiscale modeling of materials is to keep the consistency of the interfaces of different scales. In our coupled atomistic and continuum-based simulation, this consistency is ensured by applying compatible displacement fields of a screw dislocation in an infinitely large bimaterial^27^. Figure 4 shows the comparison of the distributions of stress components *σ*_*xz*_ and *σ*_*yz*_ obtained by using the atomistic method and the analytical results based on the anisotropic elasticity theory (with the elastic constants calculated from the EAM potentials^33^,^34^ used in the atomistic simulation, see Supplementary Table S1). Contours about the detailed differences between the numerical atomic displacements and stresses and the analytical ones are shown in Supplementary Information 2. It can be seen that these two methods can give almost identical stress field especially at the region of low stresses, so the continuity of stresses at the interface of the atomic region and the continuum region, and thus accuracy of this coupling method, can be ensured. It should be mentioned that, for problems where the analytical solutions are not available (e.g., a curved dislocation in a bicrystal system), the compatibility of displacements and stresses obtained with different methods can be ensured in a numerical way, such as an improved CADD method^26^.

The force exerted on a screw dislocation due to the existence of a TB in front of it can be derived with a welded bicrystal model^27^ based on the anisotropic linear elasticity theory:


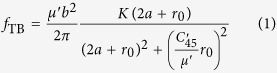


where *r*_0_ is the cutoff radius of the dislocation core (a factor of 1/2 is missing in the image-force formula in ref. 27), 
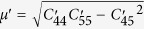
, 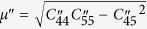,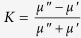, 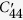, 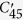, 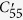 and 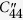, 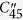, 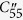 are the elastic constants for the matrix and the twin, respectively, with respect to the current coordinate system, and *a* is the distance from the dislocation to the TB. Due to the symmetry of matrix and twin, we have 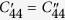, 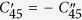 and 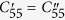. Then 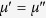 and they are expressed as *μ* for simplicity, thus *K* = 0 and *f*_TB_ = 0, that is to say, from the point view of anisotropic linear elasticity theory, the intrinsic interaction force between the TB and the screw dislocation vanishes. However, this conclusion should be taken carefully, as the linear elasticity assumption does not hold in the vicinity of the dislocation core.

The coupled simulation takes the nonlinear effect into consideration, and its accuracy is already verified by the examination of the stresses. The results in Fig. 1c show that for a screw dislocation moving 1500 Å away from the TB, the energy variation is less than 5 × 10^−4^ eV/Å for copper and 3 × 10^−3^ eV/Å for nickel. According to the anisotropic elastic theory, if the TB is replaced by a fixed screw dislocation while the other screw dislocation is located at the same position as the dislocation-TB case, the energy variation of this dislocation-dislocation interaction with respect to the change of the distance *a* between the two dislocations would be


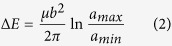


where *b* is the length of Burger’s vector of the screw dislocation. Corresponding to the simulation, the energy variation Δ*E* is 1.06 eV/Å for copper and 1.88 eV/Å for nickel. By comparison, we can see that the intrinsic long-range interaction between screw dislocations and TBs is negligible, and contributes little to the strengthening of materials.

It should be mentioned that when the distance from the TB to the dislocation is comparable to the radius of the dislocation core, as shown in Fig. 1b, the dissociation effects cannot be neglected, and the two partials dissociated from a complete one should be considered separately. In this case, the overall interaction force between a TB and a dissociated dislocation is dominated by the interaction force between the TB and the leading partial dislocation nearest to the TB if the coupling effects of the two partials are neglected. It can be inferred from the positive slope of the energy curve that the interaction between the TB and the leading partial is attractive and this attractive force should come from the edge component of the partial dislocation.

When the sample size is finite, obvious conformation force for a screw dislocation can be observed from the MEPs shown in Fig. 2. This conformation force can be attributed to the attractive image forces due to the free surfaces in the *x*-direction, the repulsion force from the TB and the overall constraint force due to fixed boundaries in the *y*-direction. Theoretical estimates show that, for a screw dislocation embedded in a finite sample, the fixed boundaries can indeed exert a constraint force to the dislocation when it departs from its equilibrium position (see Supplementary Information 4) especially when the sample height is in nano-scale. The repulsive interaction force observed in the previous work^35^ is actually a conformation force due to the rigid boundary effect in a finite large nanotwinned sample. We can infer that in ultrafine grain materials, the GBs can affect the motion of dislocations by interrupting their displacement and stress fields, and thus strengthen the material.

In samples with the free boundaries, there is no conformation force from the upper and lower boundaries. The image forces from the upper and lower boundaries are in the *y*-direction and will not influence the motion in the *x*-direction. As the dislocation is in the middle plane (from which the distances to the lower and upper boundaries are equal), the net image force is zero. As for the attractive image forces due to the free surfaces in the *x*-direction, it can be written as^28^,^35^


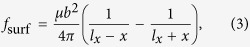


in which *l*_*x*_ the half-length of the sample and *x* the position of the dislocation. We can see that the image force from the free surfaces in the *x*-direction reduces to zero when the dislocation approaches to the TB (*x* approaches zero). Thus, the conformation force observed in the simulated samples should be mainly attributed to the intrinsic interaction between the TB when *x* approaches zero, which is shown to be negligible by the MEP curves in the free boundary cases.

The coupled simulation in an infinitely large sample and the MEP analysis in a sample of finite size with free boundaries both show that the intrinsic interaction between TBs and screw dislocations is negligible. That is to say, the TBs themselves cannot act as static barriers to repulse the approaching screw dislocations. However, extensive experimental and simulation studies^1^,^2^,^3^,^6^,^11^,^12^,^13^,^18^ have shown that TBs serve as an essential ingredient to strengthen the materials. This seeming discrepancy is solved by studying pileup behaviors of screw dislocations due to the presence of TBs. The equilibrium positions for the pileup of screw dislocations in front of a TB are shown in Fig. 3, in which the reference solutions^36^,^37^,^38^ for the pileup of screw dislocations in an anisotropic plate are also shown. In the reference solutions, the long-range force between a TB and a screw dislocations is assumed to be zero, and the effect of image forces raised from the left and right boundaries on the equilibrium positions of the pileup dislocations is neglected since it has little influence on the position due to the large sample size in the *x*-direction (see Supplementary Information 6). The simulation results agree well with the reference solution, which again verifies that the intrinsic interaction between TBs and screw dislocations is negligible.

The strengthening effects caused by TB originates from the abrupt change of the slip planes which will hinder the motion of dislocations geometrically as a high-angle GB, and the coherency of a perfect TB makes it less possible to be a source of dislocations. Though a TB itself does not repel a screw dislocation approaching it, the dislocation pinned just in front of it has elastic interactions with the following dislocations, which will cause pileup of dislocations under external stresses.

The classical Hall-Petch relationship, which describes the relationship between the flow stress and the grain size, can be represented as *σ*_*f*_ = *σ*_*Y*_ + *ad*^−1/2^, where *σ*_*f*_ is the flow stress, *σ*_*Y*_ the yield stress, d is the mean grain diameter and *α* is a parameter related to the materials^39^. To put forward this relationship, it was assumed that GBs would not repel dislocations, that is, the leading dislocation is hindered by an obstacle, such as a GB, which exerts no long-range forces. Similarly, it happens to satisfy the above-mentioned assumption in the case of TBs: the TBs themselves do not repel screw dislocations, just stop them if the stresses are not high enough for the transmission process to occur.

The good agreement between the simulation results and theoretical predictions for the equilibrium distances between pileup dislocations suggests that the TB strengthening should be well characterized by the conventional Hall-Petch relationship^39^, which is similar to the experimental observations^1^,^25^. That is, the yield strength for ultrafine-grained copper varies with twin thickness in the same manner as with grain size for nanocrystalline copper with incoherent GBs, and they both follow the empirical Hall-Petch relationship (*d*^−1/2^ dependence). It should be pointed out that if the twin lamella is too thin for a pileup to form, the stress needed for a dislocation to come across the TB will be higher than the critical stress needed to activate other deformation mechanisms, such as the migration of TBs^14^,^40^.

The equivalent grain size is reduced by the existence of TBs, and this will effectively hinder the pileup of dislocations and hence the stress concentration. When the TB spacing is on the nano-scale, the dislocation pileup is hard to occur and other deformation mechanism, such as detwinning or threading dislocations, may be operative^40^,^41^,^42^.

In summary, combined atomistic simulations and anisotropic continuum theoretical analyses reveal that the intrinsic interaction between TBs and screw dislocations is negligible. The repulsive force as demonstrated in many previous studies should be a conformation force attributed to the boundary effect in finite size samples rather than the intrinsic interaction force between perfect TBs and screw dislocations. However, the TBs can impede the motion of the screw dislocation as a result of the transformation of the slip planes across the TBs and thus the TBs and impeded dislocations can serve as a whole to repel subsequent dislocations and lead to the pileup behaviors. These results imply that the strengthening effect of TBs is mainly governed by the kinematic blocking at an earlier stage and is then driven by the kinetic interaction after accommodating some dislocations in the pileups. Moreover, the equilibrium distances between pileup dislocations are consistent with the elasticity theory predictions, which suggest that the Hall-Petch type strengthening model should be applicable to describe the TB strengthening. These findings provide new insights to understand the mechanism of TB strengthening effect and are vital for the construction of related theoretical models. It should be noted that the screw dislocations are chosen as a simple case to study the dislocation-TB interaction, and dislocations with other characters also exist in nanotwinned materials and their interactions with TBs will be studied in the future.

## Methods

### Details on the coupled atomistic and continuum-based analysis

In the coupled analysis, the geometrical size of the core atomic region is 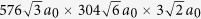 (or 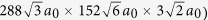, in which *a*_0_ is the lattice constant of the material, and the corresponding number of atoms is 12,607,488 (or 3,151,872). The results shown in Fig. 1a were calculated with the large cell, and the small cell model can also provide satisfactory results as shown in Supplementary Information 1. The cell size is much larger than the cutoff radius of the dislocation core. Periodic boundary condition is applied in the *z*-direction. Cells with different dislocation-TB distances *a* are constructed and the dislocation is always kept at the center of the simulation cell in order to make the most distorted part near the core of the dislocation reside in the atomistic region, as the classical linear elasticity theory is not applicable for severe deformation. The atoms in the surrounding region I are fixed with the prescribed displacements. The thickness of the region I is larger than the cut-off radius defined in the atomic EAM potentials^33^,^34^, so the surface effect can be avoided. All the atomistic simulations are performed with the open source molecular dynamics package LAMMPS^43^ and all the stress contours are drawn with the visualization tool OVITO^44^.

### Minimum energy path analysis in a finite sample

A bicrystal model is used in the MEP analysis, as shown in Fig. 1a. Periodic boundary condition is applied in the *z*-direction, and the left and right boundaries are set free, while fixed boundary condition is applied in the *y*-direction. The system sizes are 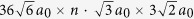 with *a*_0_ being the lattice constant of nickel and *n* varying as 38, 48 or 78 to demonstrate the size effect. The EAM potential for nickel^33^ is used to describe the interatomic interaction. Since the NEB calculation requires two end states along the MEP as inputs, stable dislocation-free and post cross-slip states are constructed (see Supplementary Information 3) and taken as the first and the last state, respectively. An elastic band consisting of 48 replicas (i.e., intermediate states) connected by springs is then created according to these two states. The stiffness constants of springs connecting neighboring replicas are chosen to be 10.0 eV/Å^2^ and the convergence criterion for the force on each atom is set to be 0.01 eV/Å.

### Pileup of screw dislocations in front of a twin boundary

Since the pileup group consists of several extended screw dislocations, a large simulation cell with size of 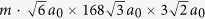 is adopted, in which *m* = 456 or 528 according to the number of pileup dislocations in the cell such that the attractive image force from the free boundaries in the *x*-direction can be neglected. The stress component *σ*_*yz*_, which is necessary to form the pileup configuration, is imported by applying additional forces in the *z*-direction to several layers of atoms at the two surfaces perpendicular to the *y*-direction. The whole system is then relaxed by alternative energy minimization based on molecular statics and dynamical evolution under the NVE ensemble (see Supplementary Information 5).

## Additional Information

**How to cite this article**: Zhang, J. *et al*. Twin Boundaries merely as Intrinsically Kinematic Barriers for Screw Dislocation Motion in FCC Metals. *Sci. Rep.* **6**, 22893; doi: 10.1038/srep22893 (2016).

## Supplementary Material

Supplementary Information
