# Supplementary material for: Twin Boundaries merely as Intrinsically Kinematic Barriers for Screw Dislocation Motion in FCC Metals
Source: Sci Rep. 2016 Mar 10;6:22893. doi: 10.1038/srep22893 (PMC4790633; doi:10.1038/srep22893)
Supplement: Supplementary Information [file srep22893-s1.pdf]

# Supplementary Information for:

# Twin Boundaries merely as Intrinsically Kinematic Barriers for Screw Dislocation Motion in FCC Metals

Jiayong Zhang[1], Hongwu Zhang[1], Hongfei Ye[1] & Yonggang Zheng[1,*]

[†] State Key Laboratory of Structural Analysis for Industrial Equipment, Department of Engineering Mechanics, Faculty of Vehicle Engineering and Mechanics, Dalian University of Technology, Dalian 116024, P. R. China

[*] Correspondence and requests for materials should be addressed to Y.G.Z. (email: zhengyg@dlut.edu.cn)

# 1. Energy curved with a small cell in the coupled analysis

In the coupled analysis, the core atomic region is simulated with two different cell sizes to check the dependence of the results on the size of the core region. The results calculated with the larger cells are shown in Fig. 1 in the main text, and the results from the smaller cells are shown in Fig. S1.

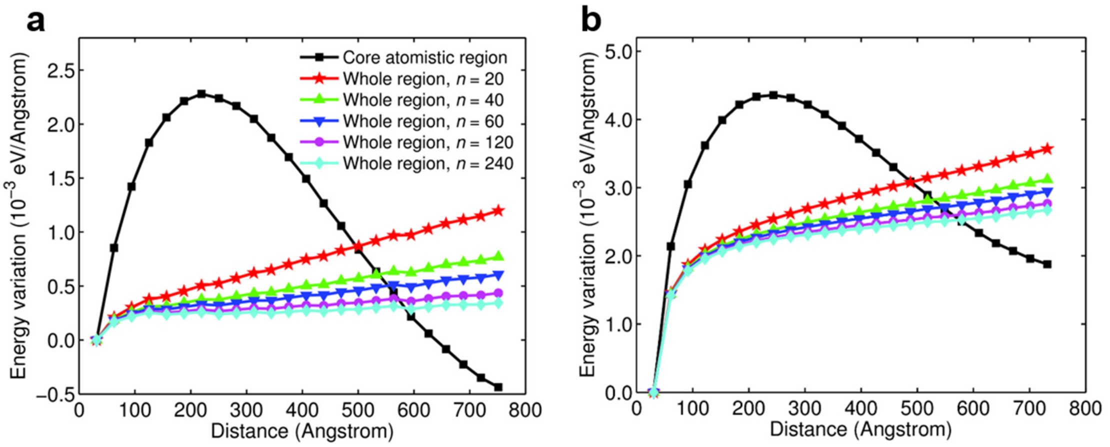


**Figure S1. The energy variation of the system as a screw dislocation moving away from the TB**. **(a)** copper and **(b)** nickel. The lines with black square marks represent the results of the inner region II calculated with the atomistic method, while others show the sum of the energy of the inner atomic region II and the surrounding continuum region I with different thicknesses.

The results calculated by using a smaller region are similar to those with a region twice larger in the $x$ and $y$ directions. This indicates that the deformation at the outer boundaries of the core atomic region is small enough to satisfy the linear elasticity assumption and the coupling method is valid.

## 2. Differences of displacements and stresses between simulation results and theoretical solutions

Due to the severe distortion near the dislocation core, the stresses in the core region calculated by atomistic simulation deviate a lot from the solutions derived with the classical anisotropic elastic theory. However, it is expected that these two methods can give consistent stresses in the region far from the dislocation core and thus a coupling atomistic – continuum method can be adopted to predict reasonably the dislocation – twin boundary interaction. To ensure the accuracy of the coupling method, it is necessary to check the consistency of the stresses across the interface between the atomic region II and the continuum region I. An alternative way is to check the consistency between the far-field stresses in region II calculated by using atomistic simulation and those derived with the classical elasticity theory. For ease of observation, only the differences of the stresses between the atomistic results and the analytical references in the region far away from the dislocation core are presented in Fig. S2.

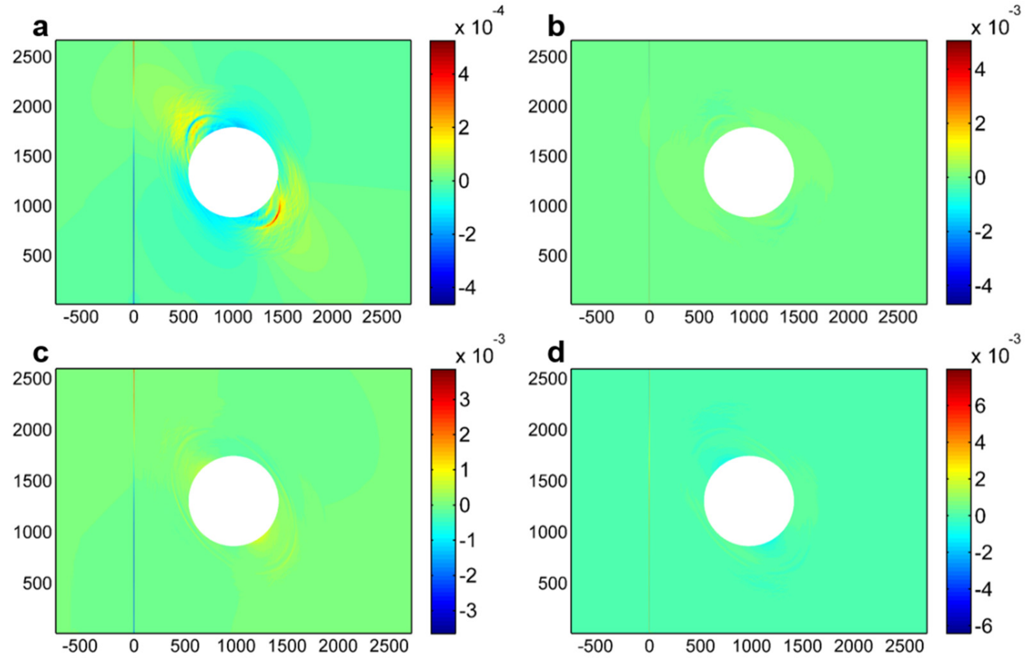


**Figure S2. Contour of the difference of the stresses between the atomistic results and the analytical references in the region far from the dislocation core. (a)** $\sigma_{xz}$ for copper, **(b)** $\sigma_{yz}$ for copper, **(c)** $\sigma_{xz}$ for nickel, **(d)** $\sigma_{yz}$ for nickel. The maximum differences are located near the TB. The stress values in the contour plots are in the unit of GPa and the position is in Angstrom.

It can be observed that the maximum differences are located near the TB. According to the elastic theory, $\sigma_{xz}$ should be continuous and $\sigma_{yz}$ is expected to be discontinuous across the TB. However, atomically sharp jump of the stresses is seen near the TB in atomistic simulations as shown in the detailed plots of the atomic stresses near the TB in Fig. S3.

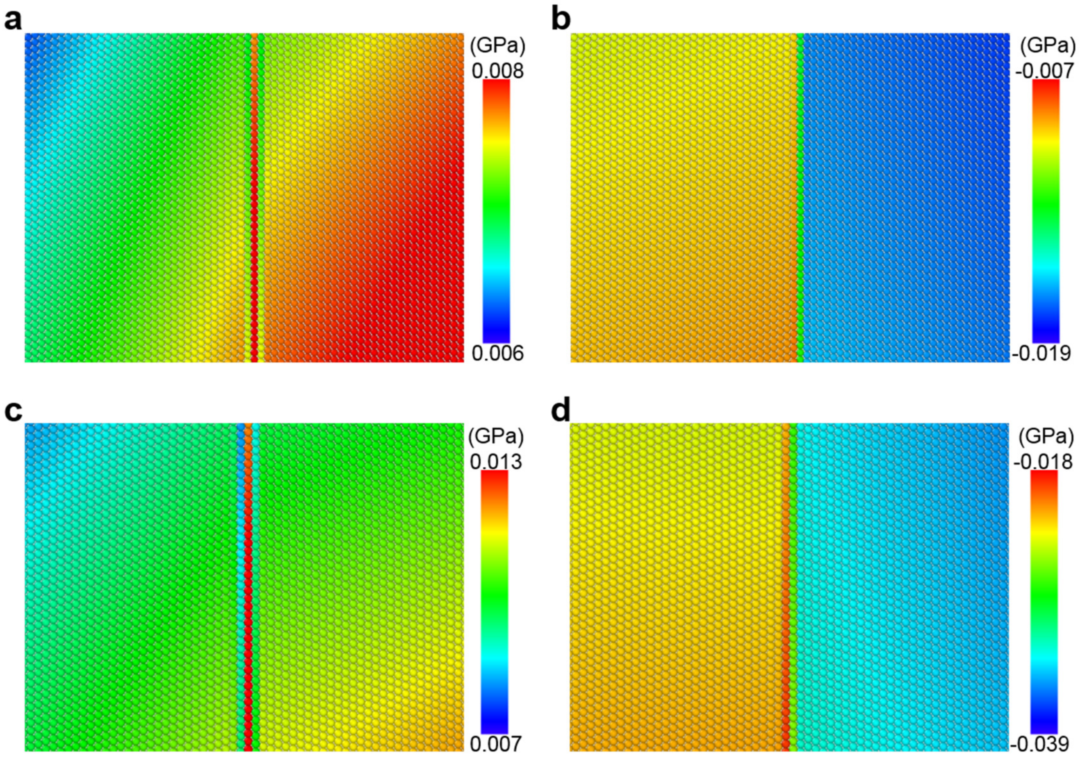


**Figure S3. The contour of the atomic stresses near the TB in copper/nickel: (a)** $\sigma_{xz}$ for copper, **(b)** $\sigma_{yz}$ for copper, **(c)** $\sigma_{xz}$ for nickel, **(d)** $\sigma_{yz}$ for nickel**.**

The irregular jump of stresses is confined within about 3 to 5 atomic planes parallel to the TB, and stresses far away from the TB are not affected. It is the overall distribution of the stresses that affect the motion of a dislocation far away from the TB, rather than the short-range irregular jump.

Stresses far away from the TB and the dislocation core agree well with the analytical solution based on the elasticity theory, as shown in Fig. S4, in which the stress differences near the TB and dislocation core regions are removed.

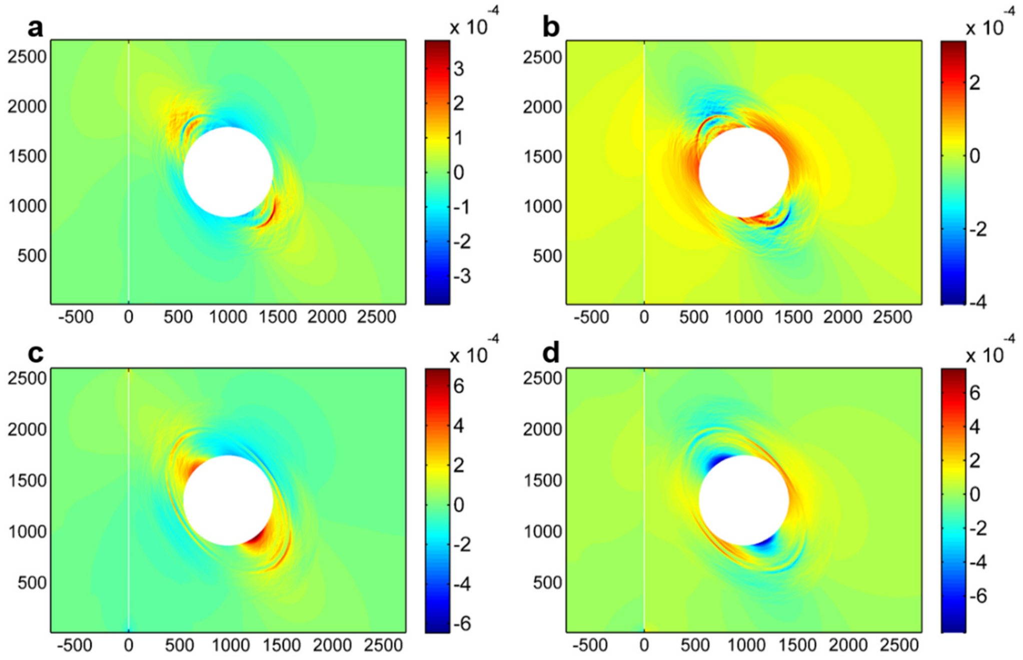


**Figure S4. Contour of the difference of the stresses between the atomistic results and the analytical references without displaying the data near the TB. (a)** $\sigma_{xz}$ for copper, **(b)** $\sigma_{yz}$ for copper, **(c)** $\sigma_{xz}$ for nickel, **(d)** $\sigma_{yz}$ for nickel. Three atomic planes in the vicinity of the TB are removed when displaying the differences. The stress values in the contour plots are in the unit of GPa and the position is in Angstrom.

It can be clearly seen that the differences of the stresses are typically less than $0.1\ \mathrm{MPa}$ near the interface between the atomic region and the continuum region. The magnitudes of these differences are small enough to ensure a good accuracy of the coupling method.

## 3. Construction of end states for the NEB analysis

The Nudged Elastic Band (NEB) calculation requires two end states (i.e., the first

and the last states) along the minimum energy path (MEP) as inputs. The first state is a dislocation-free cell and the last state is constructed as follows.

First, as shown in Fig. S5, an initial screw dislocation is introduced at the position $x_1$ (i.e., the approximate position of the dislocation core) by applying a displacement field, which is discontinuous across the slip plane, to the region $x < x_1$ according to the Burger's vector of the dislocation. Actually, based on the energy criterion, a complete screw dislocation will dissociate on the glide plane into two partial dislocations with a narrow stacking fault ribbon connecting them. To make sure that the dislocation dissociates on the central glide plane: $[01\bar{1}]/2 \rightarrow [12\bar{1}]/6 + [\bar{1}1\bar{2}]/6$ as shown in Fig. 2a in the main text, the two partials of the dislocation are created sequentially by applying displacement which is discontinuous across the central glide plane and approaches to zero at the surface layers perpendicular to the $y$-axis. That's to say, to create the leading partial, the following discontinuous displacements are applied, $\boldsymbol{u}(x < x_1; y, z > 0) = -\boldsymbol{u}(x < x_1; y, z < 0) = \frac{1}{2}\boldsymbol{\delta B} \cdot \frac{h-|y|}{h}$. While to create the trailing partial, the discontinuous displacements are $\boldsymbol{u}(x < x_1; y, z > 0) = -\boldsymbol{u}(x < x_1; y, z < 0) = \frac{1}{2}\boldsymbol{A\delta} \cdot \frac{h-|y|}{h}$, where $\boldsymbol{\delta B}$ and $\boldsymbol{A\delta}$ are the Burgers vectors of the leading and trailing partials (schematic plots are shown by the Thompson tetrahedron in Fig. 2a in the main text), respectively, $h$ is half of the distance between the top and bottom surface layers of the sample. The discontinuous displacements are applied gradually in 10 load-steps. In each load-step, the top and bottom surface layers and the parts ($x < \bar{x}$) with prescribed displacements are hold fixed and energy minimization is performed in the rest

mobile part to import the dissociated dislocation.

Then, to obtain the final state, the whole cell is uniformly deformed under an applied strain $\varepsilon_{yz}$ and relaxed under the NVE ensemble with the top and bottom layers held fixed. According to the Peach-Koehler formula, the dislocation is driven forward and impinged on the TB. If the applied strain $\varepsilon_{yz}$ is large enough, the dislocation will cross-slip into the twin part, i.e., the position $x_2$. The whole cell is then relaxed with the top and bottom layers held fixed, and is taken as the last state. It is necessary to point that in the NEB calculation, the first and the final states actually run common single-point energy minimization without being influenced by neighboring states, so a perfect energy minimization with proper boundary conditions is not required when constructing the end state. In the NEB calculations with the bottom and top layers set free, the end state is the same as that in the NEB calculations with the bottom and top layers held fixed.

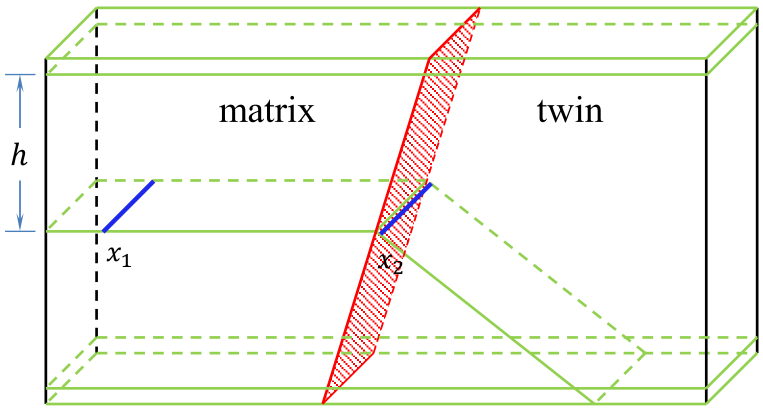


**Figure S5. Schematic representation of the simulation cell**. The last state of the NEB calculation is constructed as follows: an initial dislocation is introduced at $x_1$ in the matrix and then driven to position $x_2$ in the twin part.

## 4. Constraint force due to the fixed boundaries

Let's consider the sample shown in Fig. S6. The sample is constructed first as an infinitely large sample containing a straight dislocation along the $z$-direction with the boundary set to be free. Thus the dislocation can induce a displacement field $w(x, y)$ when the sample is fully relaxed in an equilibrium state. Then, the top ($y > h$) and bottom ($y < -h$) regions are fixed while keeping the displacements in these two regions as these induced by the dislocation. For simplicity, the initial position of the dislocation in the $xy$ plane is located at point $O$, i.e., $(0,0)$, which coincides with the origin of the coordinate system. The corresponding displacement field is denoted as $w_{(0,0)}(x, y)$, in which the subscripts $(0,0)$ represents that the dislocation is located at $(0,0)$.

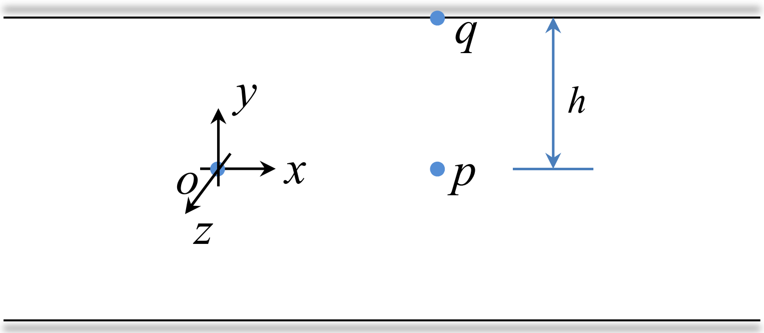


**Figure S6. Schematic representation of the motion of a dislocation in a surface-fixed plate.**

To demonstrate the constraint effect of the fixed boundaries on the dislocation, we move the dislocation to another positon $p(x_p, 0)$ with two fixed boundary

regions and estimate the force to the dislocation due to the boundaries. In this configuration, the expected equilibrium displacement field should be $w_{(x_p,0)}(x,y)$. The current configuration with the dislocation located at $p(x_p,0)$ and the displacements of the bottom and surface layers being prescribed as $w_{(0,0)}(x,y)$, can be constructed in another way: first a dislocation is introduced at $p(x_p,0)$ with the boundary set to be free and is fully relaxed, and then additional displacements are applied on the bottom and top layers. The additional displacements introduced are $w_{(x_p,0)}(x,h) - w_{(0,0)}(x,h)$. The additional displacement will cause additional shear strain $\gamma_{yz}$, which is assumed to be constant across $[-h,h]$ as a rough estimate. Thus, the additional strain is $\bar{\gamma} = \alpha \frac{w_{(x_p,0)}(x,h) - w_{(0,0)}(x,h)}{h}$, in which $\alpha$ is correction factor (here $\alpha = 1$ is assumed for simplicity) and the corresponding Peach-Koehler force is $f = \mu\bar{\gamma}b$, which is the same as the effects of some kind of background stress $\bar{\sigma} = \mu\bar{\gamma}$ . With the displacement solution of a screw dislocation in a surface-free isotropic plate[1] and the material parameter of copper, we can estimate the magnitude of the background stress caused by the fixed boundaries, as shown in Fig. S7.

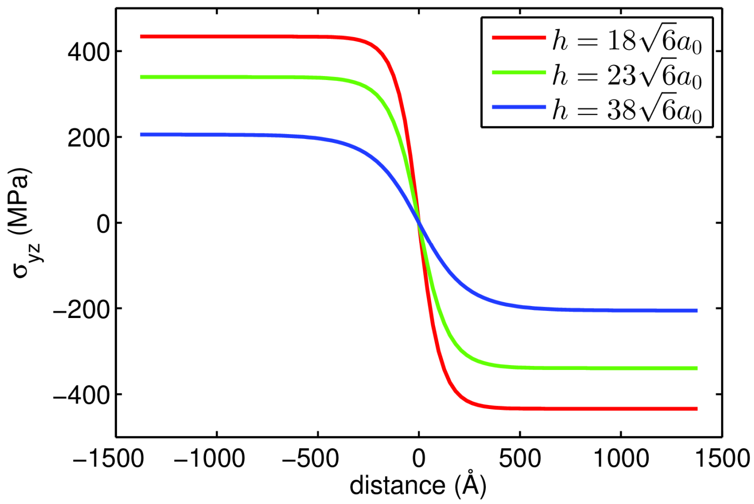


**Figure S7. The equivalent stress pinning the dislocation due to the fixed boundaries with different model heights**. The material parameters for nickel are used.

It can be seen that, as the dislocation moves away from its original equilibrium position, the Peach-Koehler force originating from the fixed boundaries, which may be mistaken as a huge resistance from the twin boundary if a nanotwinned sample was considered, increases dramatically. It can also be seen from the formula that as the height $h$ increases, the Peach-Koehler force decreases, and this is consistent with the results of the NEB calculations in the main text.

# 5. Simulation method to determine the equilibrium pile-up configuration

By repeating the operation stated in section 1 of this supplement material, with different values of $x_1$, we can get the initial configuration with many screw dislocations on the same slip plane. Then additional forces are applied to the bottom

and top layers to drive the dislocation forward. To get the equilibrium pile-up configuration with no effects of thermal fluctuations, energy minimization based on molecular statics should be performed. However, the energy minimization process tends to stop at local minima due to the discrete nature of atoms and the corresponding local Peierls barriers. So some kind of perturbation should be introduced to help the system escape local minima. The perturbation we adopted is dynamical evolution for some steps under the NVE ensemble with an initial temperature, which is high enough to help atoms overcome local Peierls barriers but not so high to make the head dislocation to enter or pass through the TB. For copper, the initial temperature is $0.1\,\mathrm{K}$ and the dynamical evolution runs $2000$ steps, while for nickel, which has a much higher unstable stacking-fault energy and thus a much higher local Peierls barrier, the initial temperature is $10\,\mathrm{K}$ and the steps needed is found by trial and error, usually more than $10000$ steps. The static energy minimization and dynamic perturbation processes are performed alternatively until the positions of all the dislocations don't change anymore. The positions of dislocations are then extracted from the equilibrium configuration with the help of Common Neighbor Analysis.[2,3]

## 6. Image force from the left and right boundaries in the dislocation pile-up sample

As shown in Fig. S8, for a plate of finite length, the image force from the left boundary can be derived by laying a dislocation with the opposite Burgers vector at

the mirror image position,[4]

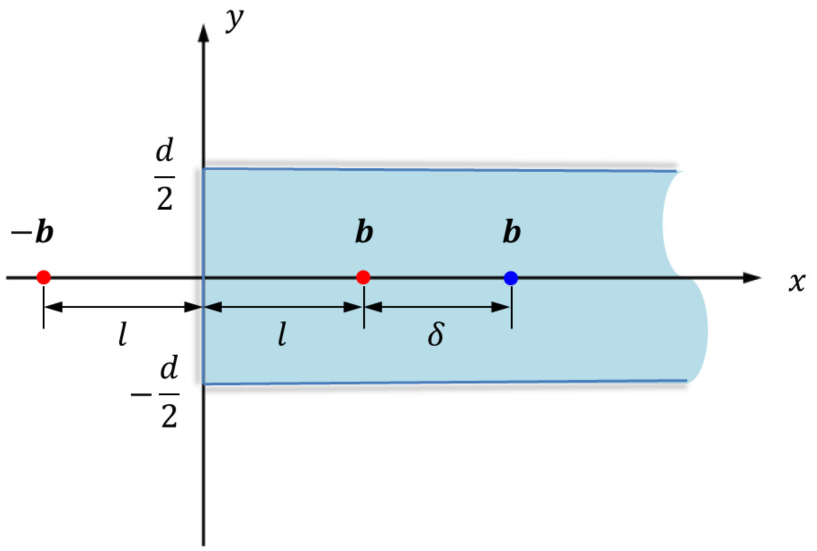


**Figure S8. Schematic representation of the finite length plate.** By placing a dislocation with opposite Burgers vector at the mirror image position, the traction-free left boundary can be simulated in the infinitely long plate.

$$f_{\text{image}} = -\frac{\mu b^2}{2d}\frac{1}{\sinh(2\bar{l})}, \tag{S1}$$

where $\bar{l} = \pi l/d$ and $l$ is the distance from the dislocation to the free boundary.

The force exerted on a dislocation from another one with a distance $\delta$ away from it is,[4]

$$f_{\text{inter}} = -\frac{\mu b^2}{2d}\frac{1}{\sinh(\bar{\delta})}, \tag{S2}$$

in which $\bar{\delta} = \pi\delta/d$. From Fig. 3a in the main text, i.e., the pile-up configuration of 5 screw dislocations caused by the TB, we can see that: (1) the distance from any dislocation to the left/right boundary is larger than the model height $d$; (2) the largest distance between two neighboring dislocations is less than $d/2$. Thus we have

$\bar{\delta} < 0.5\pi$ and $\bar{l} > \pi$ and it can be easily verified that,

$$\frac{f_{\text{image}}}{f_{\text{inter}}} = \frac{\sinh(\bar{\delta})}{\sinh(2\bar{l})} < \frac{\sinh(0.5\pi)}{\sinh(2\pi)} \approx 0.0086. \quad \text{(S3)}$$

This indicates that the contribution from the image dislocation is much less than that from the nearest neighbor dislocation, so the plate can be seen as infinitely long along the $x$-axis and it makes sense to take the solution in the case of infinite length as the reference.

**Table S1.** Materials and the corresponding elastic constants used for the atomistic simulation and theoretical analysis

| Constant | Copper | Nickel |
|---|---|---|
| $a_0$ | 3.615 Å | 3.52 Å |
| $C_{11}$ | 169.8921 GPa | 247.8473 GPa |
| $C_{12}$ | 122.5954 GPa | 147.8204 GPa |
| $C_{44}$ | 76.2057 GPa | 124.8346 GPa |
| $\mu$ | 42.4516 GPa | 79.0153 GPa |
| $C'_{44}$ | 58.6866 GPa | 99.8942 GPa |
| $C'_{45}$ | −24.7758 GPa | −35.2710 GPa |
| $C'_{55}$ | 41.1675 GPa | 74.9538 GPa |
| $C''_{44}$ | 58.6866 GPa | 99.8942 GPa |
| $C''_{45}$ | 24.7758 GPa | 35.2710 GPa |
| $C''_{55}$ | 41.1675 GPa | 74.9538 GPa |

**References**


1. Leibfried, G. & Dietze, H.-D. Zur theorie der schraubenversetzung. *Z. Für Phys.* **126,** 790–808 (1949).

2. Faken, D. & Jónsson, H. Systematic analysis of local atomic structure combined with 3D computer graphics. *Comput. Mater. Sci.* **2,** 279–286 (1994).

3. Honeycutt, J. D. & Andersen, H. C. Molecular dynamics study of melting and freezing of small Lennard-Jones clusters. *J. Phys. Chem.* **91,** 4950–4963 (1987).
4. Hirth, J. P. & Lothe, J. *Theory of dislocations*. Wiley, (1982).
